# Obstetric and offspring risks of women’s morbid conditions linked to prior anticancer treatments

**DOI:** 10.1186/s12958-016-0169-6

**Published:** 2016-07-07

**Authors:** Juan J. Tarín, Miguel A. García-Pérez, Antonio Cano

**Affiliations:** Department of Cellular Biology, Functional Biology and Physical Anthropology, Faculty of Biological Sciences, University of Valencia, Burjassot, Valencia, 46100 Spain; Department of Genetics, Faculty of Biological Sciences, University of Valencia, Burjassot, Valencia, 46100 Spain; Research Unit-INCLIVA, Hospital Clínico de Valencia, Valencia, 46010 Spain; Department of Pediatrics, Obstetrics and Gynecology, Faculty of Medicine, University of Valencia, Valencia, 46010 Spain; Service of Obstetrics and Gynecology, University Clinic Hospital, Valencia, 46010 Spain

**Keywords:** Fertility, Gestation, Gynecology, Pregnancy, Pregnancy complications

## Abstract

**Background:**

Literature shows the effects of type of cancer and/or anticancer treatment on live birth percentages and/or pregnancy and neonatal complications in female cancer survivors. However, studies analyzing the obstetric and offspring risks of the morbid conditions associated with previous anti-cancer treatments are missing. The present review aims to uncover these risks.

**Methods:**

A literature search based on publications up to March 2016 identified by PubMed and references cited in relevant articles.

**Results:**

The morbid conditions associated with prior anticancer treatments including chemotherapy, radiotherapy, surgery, and/or hematopoietic stem-cell transplant may induce not only obstetric and neonatal complications but also long-term effects on offspring. Whereas some risks are predominantly evidenced in untreated women others are observed in both treated and untreated women. These risks may be superimposed on those induced by the current women’s trend in Western societies to postpone maternity.

**Conclusions:**

Medical professionals should be aware and inform female cancer survivors wishing to have a child not only of the short- and long-term risks to themselves and their prospective offspring of previous anticancer treatments, fertility-preservation technologies, and pregnancy itself, but also of those risks linked to the morbid conditions induced by prior anticancer treatments. Once female cancer survivors wishing to have a child have been properly informed about the risks of reproduction, they will be best placed to make decisions of whether or not to have a biological or donor-conceived child. In addition, when medical professionals be aware of these risks, they will be also best placed to provide appropriate treatments before/during pregnancy in order to prevent or alleviate the impact of these morbid conditions on maternal and offspring health.

## Background

The recent advances in anticancer treatments including chemotherapy, radiotherapy, surgery, and/or hematopoietic stem-cell transplant have increased percentages of remission and survival after treatment (for review, see Tschudin and Bitzer [1]). Despite these improvements, anticancer treatments still represent an immediate threat to health as well as later health complications clinically evidenced years or even decades after completion of therapy. In fact, it is estimated that approximately two thirds of childhood cancer survivors experience at least one chronic medical problem. The other one third suffers from severe or life-threatening complications 30 years after diagnosis of their primary cancer, mostly due to adverse cardiovascular events, pulmonary dysfunction, or second malignancies including leukemias and a variety of solid tumors involving the thyroid gland, breast, cervix, corpus of the uterus, and ovaries (for reviews, see Barnes and Chemaitilly [2] and Travis et al. [3]).

Another important issue linked to anticancer treatments is the temporary or permanent loss of fertility (for review, see Knopman et al. [4]). We should note that most female cancer survivors desire to have biological children and many of them, especially childless women, may feel cancer-related infertility as an emotionally distressing and devastating health problem [5]. This distress, however, may be attenuated if female cancer patients were properly informed about the risks to fertility of anticancer therapies and offered fertility preservation options prior starting any treatment [6]. Indeed, a number of medical approaches to preserve fertility before treatment begins are being currently developed and implemented. These approaches include the use of gonadotropin releasing hormone analogs (GnRHas) for ovarian suppression during chemotherapy, fertility-sparing surgery, transvaginal immature oocyte retrieval and subsequent in-vitro maturation, oocyte cryopreservation for future in-vitro fertilization (IVF), cryopreservation of embryos after either IVF or intracytoplasmic sperm injection, and ovarian tissue banking for future orthotopic or heterotopic auto-transplantation, xeno-transplantation into immunodeficient animals, or in-vitro follicular maturation and IVF (for reviews, see West et al. [7], Dittrich et al. [8], Smyth et al. [9], and Lambertini et al. [10]). Of note, some of these procedures including ovarian tissue cryopreservation [11], in-vitro maturation [12], and ovarian suppression during chemotherapy with GnRHas [13] are still nowadays classified as experimental/investigational. Consequently, they should not be represented or marketed to patients as established or routine medical procedures [14]. They should be offered to patients only in a research setting with institutional review board oversight [15].

In addition to the risk to fertility, female cancer patients should be informed before starting any anticancer treatment about the potential short- and long-term risks of anticancer therapy and fertility-preservation practices including the risk posed by fertility treatment and the possibility of reintroducing malignant tumors cells after transplantation of cryopreserved ovarian tissue [15]. Cancer patients should know that chemotherapy and radiotherapy have the potential to induce germ cell mutations that may lead to congenital anomalies and/or genetic disease in the next generation, particularly in those cancer survivors who have not undergone a previous fertility preservation procedure. They should be informed about this possibility despite literature shows that neither chemotherapy nor radiotherapy is associated with (1) germline minisatellite mutations in survivors of childhood and young adult cancer [16]; and (2) single gene disorders, chromosomal defects, mitochondrial DNA mutations, altered sex ratio (suggesting no increased incidence of X-linked mutations), congenital abnormalities or adventitious cancer in offspring [17–19] (for reviews, see Knopman et al. [4], Hudson [20], Lawrenz et al. [21], and Nakamura et al. [22]). Cancer patients should know that the reported absence of effect of anticancer therapies on offspring genetic/chromosomal/congenital anomalies is likely due, at least in part, to the strong selection against most of the common chromosomal abnormalities present during pre- and post-implantation embryo/fetal development [23]. Accordingly, most embryos/fetuses with chromosomal anomalies may be lost before or shortly after implantation, even before women are aware that they are pregnant. Not surprisingly, literature shows that female cancer survivors without a prior fertility preservation procedure are substantially less likely to achieve a pregnancy and to have live births than their siblings or the general population (for reviews, see Knopman et al. [4] and Lawrenz et al. [21]). In addition, most birth defects are multifactorial in origin with clear interactions among genetics, epigenetics, maternal hormonal levels, and environmental exposures (e.g., medications, folate levels, nutrition, obesity, smoking, alcohol, pollutants, etc.) (for review, see Webber et al. [24]). This multifactorial origin of birth defects may dilute any existing association of anticancer therapies on offspring congenital anomalies. Notwithstanding, circumstantial evidence suggests that human immature resting oocytes compared with mouse oocytes are relatively resistant to radiation, not only in terms of cell killing but also in terms of induction of mutations (for review, see Nakamura et al. [22]).

After treatment and remission, female survivors wishing to have a child should be aware of other biological risks not only to themselves but also to their prospective offspring before making the decision to reproduce, irrespectively of whether they previously used fertility-preservation technologies or not. In particular, the potential risks posed by pregnancy on cancer recurrence (especially in breast cancer, endometrial cancer, and malignant melanoma), the difficulty in detecting cancer during pregnancy (particularly in breast cancer and endometrial cancer), and transmission of hereditary cancer syndromes (for review, see Matthews et al. [25]).

Although literature evidences the effects of type of cancer and/or anticancer treatment on live birth percentages and/or pregnancy and neonatal complications (for reviews, see Knopman et al. [4], Hudson [20], and Lawrenz et al. [21]), studies showing the obstetric and offspring risks of the morbid conditions associated with previous anti-cancer treatments are missing. In order to fill this gap, the present review aims to uncover and highlight the obstetric and offspring risks of the morbid conditions associated with previous anti-cancer treatments.

## Methods

A literature search based on publications up to March 2016 identified by PubMed database searches using the following search terms: female cancer survivors, obstetric and neonatal risks, long-term risks, offspring, hyperprolactinemia, hypopituitarism, hypothyroidism, hyperthyroidism, primary ovarian insufficiency, obesity, overweight, hyperglycemia, insulin resistance, metabolic syndrome, diabetes mellitus, cardiovascular disease, obstructive lung disease, restrictive lung disease, decreased pulmonary diffusion capacity, chronic kidney disease, chronic hypertension, uterine damage, and low bone mineral density. In addition, a hand search was done to explore the references cited in the primary articles. Only articles (whenever possible systematic reviews and meta-analyses) published in English were included.

## Results

Table 1 shows the potential obstetric and offspring risks of morbid conditions associated with prior anticancer treatment. Note that whereas some risks are predominantly evidenced in untreated women others are observed in both treated and untreated women. For instance, the increased risk of seizure (neonatal seizure, febrile seizure, and epilepsy), autism spectrum disorders, and attention-deficit hyperactivity disorder in offspring associated with maternal hypothyroidism or hyperthyroidism is mainly observed when the mother is first time diagnosed and treated for thyroid dysfunction after birth of the child, not before/during pregnancy (for review, see Andersen et al. [26]). Such a circumstance suggests that diagnosis and treatment of thyroid dysfunction before/during pregnancy may prevent or alleviate the effects of maternal thyroid disease on early brain development (for review, see Andersen et al. [26]). Likewise, (1) untreated hyperprolactinemia may be a risk factor for ectopical pregnancy [27]; (2) uncontrolled overt hyperthyroidism is associated with increased risk of thyroid storm, maternal congestive heart failure, miscarriage, stillbirth, preterm delivery, pre-eclampsia, low birth weight, intrauterine growth restriction, and fetal/neonatal thyroid dysfunction (for review, see Pearce [28]); (3) untreated euthyroid pregnant women with detectable thyroid autoantibodies display higher risks of miscarriage and preterm delivery than treated women (for systematic review, see Thangaratinam et al. [29]); and (4) tight glycemic control as well as dietary antioxidant supplementation during the preconception period and during the first trimester of pregnancy can prevent diabetes-associated birth defects and pregnancy complications (for review, see Ornoy et al. [30]). Notwithstanding, the level of glycemic control and glycemic threshold in pregnancy for preventing offspring complications later in life is still unknown (for review, see Hiersch and Yogev [31]).

**Table 1:** Potential obstetric and offspring risks of morbid conditions associated with prior anticancer treatments including chemotherapy, radiotherapy, surgery, and/or hematopoietic stem-cell transplant

Morbid conditions associated with prior anticancer treatments	Potential obstetric and neonatal risks^a^	Potential long-term risks to offspring^a^
Endocrine disorders (for review, see Barnes and Chemaitilly [2]).		
Hypothalamic-pituitary axis: hyperprolactinemia and deficiencies in growth hormone (GH), luteinizing hormone (LH), follicle-stimulating hormone (FSH), adrenocorticotropic hormone (ACTH), thyroid stimulating hormone (TSH), and antidiuretic hormone (ADH).	- Obstetric complications are rare in hyperprolactinemic women treated or untreated with bromocriptine, although untreated hyperprolactinemia may be a risk factor for ectopical pregnancy (25 %, 4/78 vs. 5 %, 6/25 in bromocriptine-treated pregnancies (*P* = 0.017) [27].	
	- Hyperprolactinemia may lead to galactorrhea and nipple tenderness which are not suitable for breastfeeding (for review, see Du et al. [40]).	
	- Maternal hypopituitarism is associated with increased risk of abortion during early gestation, anemia, pregnancy-induced hypertension, placental abruption, premature birth, cesarean birth, and postpartum uterine inertia leading to postpartum hemorrhage (for review, see Du et al. [40]).	
- Thyroid gland: primary hypothyroidism with elevated plasma TSH levels and either normal or low levels of free thyroxine (T_4_), hyperthyroidism, or autoimmune induced thyroid disease (note that the presence of thyroid antibodies does not necessarily lead to hypothyroidism in the context of hematopoietic stem-cell transplant).	- Pregnant women with subclinical hypothyroidism (elevated plasma TSH levels and normal free T_4_) have increased risk of miscarriage, gestational diabetes, preterm delivery, and placental abruption (for review, see Pearce [28]). Likewise, maternal overt hypothyroidism (elevated plasma TSH levels and low free T_4_) is associated with miscarriage, stillbirth, gestational hypertension, preterm delivery, and low birth weight (for review, see Pearce [28]).	- Maternal subclinical or overt hypothyroidism is associated with decreased child intelligence (for review, see Pearce [28]). Maternal hypothyroidism is also associated with increased risk of neonatal seizure [adjusted hazard ratio (HR):1.08, 95 % confidence interval (CI): 1.02-1.15], febrile seizure (adjusted HR: 1.21, 95 % CI: 1.10-1.32), and epilepsy (adjusted HR: 1.22, 95 % CI: 1.06-1.40); childhood autism spectrum disorders (adjusted HR: 1.30, 95 % CI:1.11-1.53); and adolescence and young adulthood psychiatric disorders (adjusted HR for use of antipsychotics: 1.22, 95 % CI: 1.03-1.44; and adjusted HR for use of anxiolytics: 1.23, 95 % CI: 1.03-1.48) (for review, see Andersen et al. [26]).
	- Pregnant women with subclinical hyperthyroidism is not associated with adverse maternal or fetal outcomes (for review, see Pearce [28]). In contrast, uncontrolled overt hyperthyroidism during pregnancy is associated with increased risk of thyroid storm, maternal congestive heart failure, miscarriage, stillbirth, preterm delivery, pre-eclampsia, low birth weight, intrauterine growth restriction, and fetal/neonatal thyroid dysfunction (for review, see Pearce [28]).	- Maternal hyperthyroidism increases the risk of epilepsy (adjusted HR: 1.20, 95 % CI: 1.09-1.32) and attention-deficit hyperactivity disorder (adjusted HR: 1.18, 95 % CI: 1.03-1.36) (for review, see Andersen et al. [26]).
	- Autoimmune hypo- or hyperthyroidism during pregnancy is associated with the same obstetrical and neonatal risks posed by non-autoimmune hypo- or hyperthyroidism (see above). In addition, euthyroid pregnant women with detectable thyroid autoantibodies display higher risk of miscarriage [in cohort studies, random effects odds ratio (OR):3.90, 95 % CI: 2.48-6.12; in case-control studies, random effects OR:1.80, 95 % CI: 1.25-2.60] and preterm delivery (in cohort studies, random effects OR: 2.07, 95 % CI: 1.17-3.68) (for systematic review, see Thangaratinam et al. [29]). Notwithstanding, treatment with levothryroxine reduces the risk of miscarriage [relative risk (RR):0.48, 95 % CI:0.25-0.92] and preterm delivery (RR: 0.31, 95 % CI: 0.11-0.90) (for systematic review, see Thangaratinam et al. [29]).	- Autoimmune hypo- or hyperthyroidism during pregnancy is associated with the same long-term risks to offspring posed by non-autoimmune hypo- or hyperthyroidism (see above). Furthermore, children of euthyroid pregnant women with elevated titers of thyroid peroxidase autoantibody (TPOAb) are at risk for transient hyperthyroxinemia one week after birth (the values of free T_4_ are, however, normalized after 15 days) [48] and impaired psychomotor development at 5 years of age (adjusted OR for the scores on the General Cognitive Scale: 10.5, 95 % CI: 3–34) (for review, see Dallas [49]). Transplacentally-transmitted TPOAb positivity at birth is also associated with increased incidence of autoimmune thyroiditis during childhood and adolescence (adjusted OR: 4.12, 95 % CI: 1.79-9.50) [50].
- Gonads: acute ovarian insufficiency (i.e., women do not experience a recovery of their ovarian function after finishing cancer treatments) or primary ovarian insufficiency (POI), previously called premature ovarian failure or premature or early menopause (i.e., women resume pubertal development or menstrual cycles after finishing cancer treatments but experience ovarian failure before the age of 40 years).	- In contrast to natural menopause, women diagnosed with POI may undergo unpredictable ovarian function leading to intermittent and unpredictable menses in 50 % of cases. Pregnancy and delivery in women with POI are unlikely and rare (for review, see Tarín et al. [41]).	
- Body composition: survivors of acute lymphoblastic leukemia and brain tumors have higher risks of obesity and overweight.	- Pregnant women with obesity have higher risk of gestational diabetes (OR: 3.76, 95 % CI: 3.31-4.28 for women with body mass index (BMI) > 29.9 kg m^−2^), gestational hypertension (4.5-8.7 times more likely than normal weight women), pre-eclampsia (pooled RR: 2.68, 95 % CI: 2.40-3.00 for women with BMI 30–34.9 kg m^−2^), antenatal (OR: 1.43, 95 % CI: 1.27-1.61) and postnatal (OR: 1.30, 95 % CI: 1.20-1.42) depression, antenatal anxiety (OR: 1.41, 95 % CI: 1.10-1.80), instrumental-vaginal (OR: 1.17, 95 % CI: 1.13-1.21) and cesarean birth (ORs from 2.01, 95 % CI: 1.87-2.15 to 2.36, 95 % CI:2.15-2.59), and surgical site infection than pregnant women of healthy weight. In addition, maternal obesity is associated with higher risk of congenital anomalies, preterm birth (adjusted OR: 1.33, 95 % CI: 1.12-1.57 for women with BMI ≥ 35 kg m^−2^), large-for-gestational-age babies (above the 90^th^ centile) (OR: 2.08, 95 % CI:1.95-2.23), miscarriage (pooled OR: 1.31, 95 % CI: 1.18-1.46 for women with BMI ≥ 28 kg m^−2^), fetal death (RR: 1.34, 95 % CI: 1.22-1.47 for women with BMI ≥ 30 kg m^−2^), stillbirth (adjusted OR: 1.6, 95 % CI:1.35-1.95 for women with BMI ≥ 30 kg m^−2^), birth over 41–42 weeks’ gestation, induction of labor, failure to progress in labor, post-partum hemorrhage, longer duration of hospital stay, neonatal intensive care unit admission, lower breastfeeding initiation incidence (ORs from 1.19 to 3.65), and shorter breastfeeding duration (HRs from 1.24 to 2.54) [42] (for systematic review, see Marchi et al. [43]).	- Maternal obesity is associated with cardiovascular and metabolic disorders, obesity, childhood asthma and wheezing, cognitive development deficits, attention-deficit hyperactivity disorder, cancer, and greater all-cause mortality (for review, see Wilson and Messaoudi [51]).
- Glucose metabolism: insulin resistance, metabolic syndrome, and diabetes mellitus.	- Maternal diabetes increases the risk of congenital anomalies affecting any developing organ system, although cardiovascular and neural tube defects are among the most frequent anomalies. Furthermore, it increases the risk of pre-eclampsia, preterm delivery, fetal macrosomia, low birth weight, and perinatal mortality [42].	- Children born to diabetic mothers tend to be overweight and taller, and have increased incidence of gross and fine motor abnormalities, attention deficit hyperactivity disorder, learning difficulties, likely autism spectrum disorder, and metabolic morbidity later in life (for reviews, see Hiersch and Yogev [31] and Ornoy et al. [30]).
	- Pregnant women with insulin resistance display increased risk of miscarriage, pre-eclampsia, and fetal macrosomia, especially in pregnancies complicated by type 1 diabetes mellitus [44] (for review, see Gutaj et al. [45]). Moreover, maternal pregestational type 2 diabetes mellitus (insulin resistance) is associated with a higher prevalence of birth defects in offspring [adjusted prevalence ratio (PR): 4.51, 95 % CI: 2.46-8.29] including cardiovascular, genitourinary, and musculoskeletal defects [46].	
	- Women with metabolic syndrome have a higher risk of pre-eclampsia, preterm delivery or low birth weight (for review, see Malek [47]).	- Children of mothers with metabolic syndrome are likely to develop metabolic syndrome and cardiovascular disease (for review, see Malek [47]).
Adverse cardiovascular events (for reviews, see Travis et al. [3] and Ewer and Ewer [52]).		
- Left ventricular systolic dysfunction, cardiovascular disease (pericardial disease, coronary artery disease, carotid artery occlusive disease, premature valvular calcification and dysfunction, conduction abnormalities, and cerebrovascular disease), cardiac arrhythmias, and cardiac ischemia.	- Pregnant women suffering from heart diseases have increased risk of maternal cyanosis, risk of bleeding due to anticoagulation treatment, placental hypoperfusion and/or cyanosis, fetal growth restriction, miscarriage, stillbirth, and prematurity (for review, see Emmanuel and Thorne [34]).	
Pulmonary dysfunction in childhood cancer survivors, particularly in women treated with pulmonary-toxic chemotherapy and survivors treated with more than 20 Gy chest radiation [53].		
- Restrictive lung disease with reduced lung volumes as a result of either decreased lung parenchyma or changes to the chest wall that may restrict lung parenchyma growth.	- The ability to increase the minute ventilation during pregnancy may be limited in pregnant women with restrictive lung disease. Consequently, they may have difficulty meeting the increased oxygen demands of late pregnancy putting the fetus at risk of hypoxic injury [54].	- Fetal chronic hypoxia induces fetal growth restriction and programs cardiovascular, metabolic, and endocrine dysfunction in the adult offspring (for review, see Giussani et al. [56]).
- Decreased pulmonary diffusion capacity.	- Cancer survivors with decreased pulmonary diffusion capacity are more likely to report respiratory symptoms, poor physical functioning, low energy and increased fatigue than survivors without diffusion defects [53]. These effects may be exacerbated during the third trimester of pregnancy when pulmonary diffusion capacity decreases compared to non-pregnant women especially in women living at high altitude [55].	
Renal adverse effects (for systematic review, see Knijnenburg et al. [57]; for review, see Porta et al. [58]).		
- Acute kidney injury, renal insufficiency, chronic kidney disease, impaired glomerular filtration rate, proteinuria, electrolyte disturbances, impaired phosphate tubular regulation, hypertension, and thrombotic microangiopathies.	- Pregnant women with chronic kidney disease and serum creatinine levels > 1.5 mg/dL have higher risk of gestational deterioration in renal function. In addition, there is a negative linear association between maternal proteinuria and infant birth weight as well as between the severity of maternal proteinuria and degree of renal dysfunction and fetal loss (for review, see Gyamlani and Geraci [59]).	
	- Treated chronic hypertension in pregnancy is associated with increased risk of pre-eclampsia (adjusted OR: 6.0, 95 % CI: 4.7-8.8), placental disorders (adjusted OR: 2.3, 95 % CI: 1.8-3.1), gestational diabetes (adjusted OR: 2.2, 95 % CI: 1.4-3.5), threatened abortion (adjusted OR: 1.9, 95 % CI: 1.7-2.1), preterm delivery (adjusted OR: 1.5, 95 % CI: 1.3-1.8), and low birth weight (adjusted OR: 2.3, 95 % CI: 1.8-2.7) (for review, see Czeizel and Bánhidy [32]). In addition, pregnancies complicated by chronic hypertension are at increased risk of congenital abnormalities, particularly cardiac malformations (adjusted OR in treated women: 1.06, 95 % CI: 1.4-1.9; adjusted OR in untreated women: 1.5, 95 % CI: 1.3-1.7) (for review, see Bateman et al. [33]).	
Uterine damage (for reviews, see Lawrenz et al. [21], Wo and Viswanathan [60]; Wallace et al. [61], and Teh et al. [62]).		
- Total-body, flank, abdomen, pelvis or direct uterine irradiation leads to changes in the uterus including reduced uterine volume, endometrial and myometrial atrophy, uterine fibrosis, loss of elasticity of the myometrium, and reduced blood flow.	- Radiotherapy-induced structural and functional changes to the uterus may adversely affect implantation and maintenance of pregnancy increasing the risk of placental attachment disorders (placenta acreta or placenta percreta), low birth weight (RR: 1.85, p = 0.03 in patients treated with pelvic irradiation, and ORs from 3.64, 95 % CI: 1.33-9.96 in survivors after abdominopelvic radiation up to 6.8, 95 % CI: 2.1-22.2 in patients treated with high-dose (>5 Gy) radiotherapy to the uterus), small for gestational age (OR: 4.0, 85 % CI: 1.6-9.8 in patients treated with high-dose (>5 Gy) radiotherapy to the uterus), preterm delivery (OR: 3.5, 95 % CI: 1.5-8.0 in patients treated with high-dose (>5 Gy) radiotherapy to the uterus), perinatal death, and fetal malposition (for review, see Wo and Viswanathan [60]).	
	- Surgical removal of the uterine cervix in cervical cancer patients is associated with increased risk of second trimester loss (10 % of pregnancies) and premature delivery in the third trimester (19 % of pregnancies) (for review, see Knopman et al. [4]).	
Musculoskeletal deficiencies in survivors of childhood cancer (for reviews, see Wasilewski et al. [63], Barnes and Chemaitilly [2], and Wissing [64]).		
- Low bone mineral density	- A deficit in bone mineral density is associated with increased susceptibility to fragility fractures in pregnancy or the puerperium that may result from the combination of abnormal skeletal microarchitecture prior to pregnancy and increased bone resorption during pregnancy (for review, see Kovacs [65]).	
	- During pregnancy and lactation, calcium homeostasis is overwhelmingly in favor of the fetus/neonate, and the fetal/neonatal calcium levels are maintained despite or even at the expense of maternal levels and health (for review, see Done [66]).	

^a^ORs, HRs, PRs, or RRs to mothers and offspring of some morbid conditions linked to prior anticancer treatments are missing because of reviewed data come from literature narrative syntheses

On the contrary, chronic hypertension is associated with increased risk of adverse obstetrical and neonatal outcomes including pre-eclampsia, placental disorders, gestational diabetes, threatened abortion, preterm delivery, low birth weight, and congenital malformations, irrespectively of whether women are treated or not during pregnancy (for reviews, see Czeizel and Bánhidy [32] and Batemanet al. [33]). Of note, pregnant women suffering from chronic hypertension treated with antihypertensive drugs display ORs as high as 6.0 for pre-eclampsia, 2.3 for placental disorders, and 2.2 for gestational diabetes compared with control pregnant women without any type of hypertension (for review, see Czeizel and Bánhidy [32]). In addition, there are morbid conditions associated with prior anticancer therapies displaying problematic, controversial, or no treatment at all. For instance, obesity, restrictive lung disease, decreased pulmonary diffusion capacity, and uterine damage are not easily managed in clinical practice. Furthermore, many drugs prescribed for heart disease have teratogenic effects. Therefore, medication should be reviewed prior to pregnancy (for review, see Emmanuel and Thorne [34]).

Finally, we cannot ignore that maternal age at childbirth is steadily rising in many Western populations, and female cancer survivors are not an exception to this general trend [35]. The resulting obstetric and offspring risks associated with postponed maternity (for reviews, see Usta and Nassar [36], Nassar and Usta [37], and Sauer [38]) may be superimposed on those already present in cancer survivors. Importantly, the extra risks posed by delayed motherhood may not be prevented by applying fertility preservation strategies such as oocyte/embryo/ovarian tissue cryopreservation at younger ages. In fact, reciprocal ovarian transplants between young and old female mice show that the risk of congenital heart disease associated with advanced maternal age is not conferred by oocytes, but by the mother’s age [39]. Interestingly, this risk is modified by the mother’s genetic background and can be mitigated (but not eliminated entirely) by maternal voluntary (ad libitum) exercise beyond a threshold number of days before birth date, whether exercise begins at a young age or later in life [39].

## Conclusions

The present review shows that the morbid conditions associated with prior anticancer treatments including chemotherapy, radiotherapy, surgery, and/or hematopoietic stem-cell transplant may induce obstetric and neonatal complications as well as long-term effects on offspring. Of note, whereas some risks are predominantly evidenced in untreated women others are observed in both treated and untreated women. These risks may be superimposed on those induced by the current women’s trend in Western societies to postpone maternity. Medical professionals should be aware and inform female cancer survivors wishing to have a child of the short- and long-term risks to themselves and their prospective offspring irrespectively of whether they previously used fertility-preservation technologies or not. These risks not only include those associated with previous anticancer treatments, fertility-preservation technologies, and pregnancy itself, but also those linked to the morbid conditions induced by prior anticancer treatments. Once female cancer survivors wishing to have a child have been properly informed about the risks of reproduction, they will be best placed to make decisions of whether or not to have a biological or donor-conceived child. In addition, when medical professionals be aware of these risks, they will be also best placed to provide appropriate treatments before/during pregnancy in order to prevent or alleviate the impact of these morbid conditions on maternal and offspring health.

## Abbreviations

ACTH, adrenocorticotropic hormone; ADH, antidiuretic hormone; BMI, body mass index; CI, confidence interval; FSH, follicle-stimulating hormone; GH, growth hormone; GnRHa, gonadotropin releasing hormone analog; HR, hazard ratio; IVF, in-vitro fertilization; LH, luteinizing hormone; OR, odds ratio; POI, primary ovarian insufficiency; PR, prevalence ratio; RR, relative risk; T_4_, thyroxine; TPOAb, thyroid peroxidase autoantibody; TSH, thyroid stimulating hormone
